# Influence of spray stand-off distance, plasma intensity, and Ar/H_2_ ratio, on the phase composition and microstructure of bismuth-containing 45S5 bioactive glass coatings deposited by atmospheric plasma spray (APS)

**DOI:** 10.1016/j.rsurfi.2026.100898

**Published:** 2026-08

**Authors:** G.A. Clavijo-Mejía, M. Michálek, F. Kurtuldu, L. Youssef, E. Bussy, A. Denoirjean, A. Magnaudeix, D. Cabrera-German, D. Galusek, A.R. Boccaccini

**Affiliations:** a FunGlass, Alexander Dubček University of Trenčín, Študentská 2, Trenčín, 911 50, Slovakia; b Institute of Research for Ceramics–IRCER, UMR 7315, CNRS, Centre Européen de la Céramique (CEC), Université de Limoges, 12 Rue Atlantis, Limoges Cedex, 87068, France; c Departamento de Investigación en Polímeros y Materiales, Universidad de Sonora, Blvd. Luis Encinas y Rosales S/N., Hermosillo, Sonora, 83000, Mexico; d Joint Glass Centre of the IIC SAS, TnUAD, and FChPT STU, Študentská 2, Trenčín, 911 50, Slovakia; e Institute of Biomaterials, Department of Material Science and Engineering, University of Erlangen-Nuremberg, Erlangen, 91058, Germany

## Abstract

The study investigates the influence of atmospheric plasma spray (APS) parameters on the structure and chemistry of coatings produced from a bismuth-containing 45S5 bioactive glass (Bi-BG). A melt-derived glass modified with 6.3 wt % Bi_2_O_3_ was used as feedstock powder, selected for its previously demonstrated radiopacity, cytocompatibility, angiogenic, and antibacterial activity. Coatings were deposited on titanium alloy substrates using parameter combinations varying arc current intensity, Ar/H_2_ gas mixture ratio, and stand-off distance (SOD). Phase composition, morphology, and short-range network structure were examined by XRD, SEM, DSC, and Raman spectroscopy. Short SODs combined with high plasma net power reduced deposition efficiency and increased porosity, whereas longer distances favored amorphous phase retention and improved intersplat bonding. A Latin Square design of experiments revealed that SOD was the dominant factor governing hardness, porosity, and thickness, accounting for 31 – 53 % of the total variance, while arc current intensity showed a threshold effect on porosity reduction at elevated current levels. Raman spectroscopy revealed significant changes in phosphate and silicate network units, particularly in symmetric P–O–P and asymmetric Si–O–Si groups. Coatings deposited at moderate plasma net power (24.7 – 29.9 kW) and a long SOD of 120 mm exhibited interfacial hardness values of 296 – 349 HV_0.5_, porosity below 2.0 %, and thicknesses of 77 – 80 μm, while largely preserving the amorphous network and enhancing mechanical integrity at the coating–substrate interface. This first systematic investigation of APS processing parameters for bismuth-modified 45S5 bioactive glass coatings provides practical guidance for fabricating structurally stable, bioactive, and radiopaque coatings for implantable biomedical devices.

## Highlights

• First APS-fabricated bismuth-containing 45S5 bioactive glass coatings are demonstrated.
• APS parameters control amorphous phase retention, deposition efficiency, and microstructure.
• Short stand-off distance and high plasma power increase porosity and reduce deposition efficiency.
• Raman analysis reveals APS-induced changes in silicate and phosphate network structures.
• Optimized APS conditions produce dense, amorphous, mechanically stable bioactive coatings.

## 1 Introduction

45S5 Bioglass is one of the most extensively studied bioactive glasses due to its ability to bond directly to bone tissue and stimulate osteogenesis. This behavior is attributed to interfacial reactions between the bioactive glass (BG) and physiological media, leading to the formation of a hydroxycarbonate apatite (HCA) layer on the glass surface. During this process, the glass network connectivity, ionic exchange, and dissolution kinetics govern the material's biological response [[1], [2], [3], [4]]. Since the dissolution rate is closely related to the structural organization of the glass, increased crystallinity reduces dissolution, delays apatite formation, and impairs bone bonding efficiency [[5], [6], [7]]. Despite their remarkable bioactivity and cytocompatibility, silicate bioactive glasses exhibit intrinsic brittleness and low fracture toughness. As a result, their direct use in load-bearing applications is limited compared with calcium phosphate ceramics [3,8]. A practical solution lies in the functionalization of metallic implants, such as Ti and Ti6Al4V alloys, with BG coatings. This strategy overcomes the mechanical limitations of glass while maintaining its biological functionality [[9], [10], [11]]. Such composite systems aim to combine the bioactivity and rapid bone integration of the glass with the mechanical stability of the metal substrate, thereby improving implant longevity. The performance depends not only on the glass composition and bioactivity but also on the coating-substrate adhesion, microstructural integrity, porosity distribution, and residual stress development.

Among various coating techniques used to functionalize metallic surfaces, plasma-sprayed ceramic coatings have demonstrated superior fixation and enhanced bone-implant contact [11,12]. Atmospheric plasma spraying (APS) offers several advantages for 45S5 BG-based coatings, including high deposition rates, controllable thickness, strong adhesion, and suitability for complex geometries [13]. However, the high thermal energy of the plasma jet significantly influences the BG in-flight temperature, the splat formation, particle flattening, and the volatilization of thermally sensitive components such as Na_2_O and P_2_O_5_. Consequently, phase composition, porosity, and intersplat bonding are highly sensitive to APS parameters [[14], [15], [16], [17]]. Plasma net power, determined by the intensity and Ar/H_2_ ratio, together with the stand-off distance (SOD), defines the in-flight particle temperature and velocity, thereby controlling the degree of crystallinity, coating deposition efficiency, intersplat cohesion, and coating densification [14,15]. Since the coating's densification and intersplat bonding directly influence its mechanical integrity, improved adhesion can be achieved by optimizing BG particle melting and substrate interaction [18]. BG crystallization, residual porosity, and splat cohesion determine the hardness, adhesion strength, and integrity of plasma-sprayed bioactive glass coatings [17,19,20]. Enhanced compactness typically increases hardness and fracture resistance, as observed in other thermally sprayed systems [21]. Nonetheless, retaining the amorphous nature of BG while achieving sufficient densification remains a critical challenge under high-temperature APS processing [13,22].

Parallel advances in BG research have focused on incorporating functional ions to impart additional biological or physicochemical properties. The inclusion of such ions modifies network connectivity and dissolution rates, allowing tailored cellular responses. Several doped glass systems were reported to exhibit antibacterial activity while preserving their bioactivity [23,24]. Bismuth (Bi) is particularly interesting as a glass modifier due to its high atomic number, radiopacity, antibacterial features, and biocompatibility. The incorporation of Bi_2_O_3_ into the 45S5 BG network preserves the amorphous structure while modulating its dissolution behavior [25].

Our previous studies demonstrated that adding 6.3-7.2 wt % of Bi_2_O_3_ into 45S5 increased radiopacity 2.3-2.5 times without inducing crystallization. Structural analyses indicated the coexistence of [BiO_3_] and [BiO_6_] units, reflecting the dual network former-modifier role of bismuth oxide, which reduced the dissolution rate and the BG bioactivity. Moreover, Bi-containing 45S5 BGs exhibited high radiopacity, excellent cytocompatibility, vascular endothelial growth factor (VEGF) secretion, and antibacterial activity, all being desirable attributes for bone tissue applications [26,27]. Although the structure and biological performance of bulk Bi-BGs have been established, their behavior under high-temperature manufacturing processes, such as thermal spray deposition, remains unexplored. Since Bi_2_O_3_ incorporation alters the glass network, it also affects viscosity, crystallization, and volatilization under plasma conditions. Given that plasma net power and SOD determine the particle in-flight melting degree, cooling rate, and deposition efficiency, the response of Bi-BG compositions cannot be extrapolated from conventional 45S5 BG coatings [14,15,[18], [19], [20]]. A systematic statistical approach is therefore necessary to independently quantify the contribution of each processing parameter to the coating properties of this new composition. Therefore, understanding of how the APS parameters influence structure, phase evolution, and microstructure in bismuth-containing 45S5 BG coatings is essential particularly considering that Bi_2_O_3_ acts as a dual former-modifier through [BiO_3_]/[BiO_6_] units, reducing T_g_ to 509 °C and narrowing the crystallization window T_g_ – T_x_ ≈ 91 °C, while its high boiling point ∼1890 °C ensures bismuth retention across the full APS processing parameters, a thermal behavior with no direct analogue in conventional or ion-doped 45S5 BG systems. A statistical design of experiments offers an objective means to assess the individual influence of processing parameters on the resulting coating properties of this new composition.

This work investigates the effect of APS parameters on the structural, phase, and microstructure evolution of bismuth-containing 45S5 bioactive glass coatings. The influence of plasma intensity, gas ratio, and spray stand-off distance on coating, thickness, porosity, and the interface hardness of coating-substrate is analyzed through a Latin Square design of experiments. To the best of the authors’ knowledge, this study is the first to report the use of bismuth-modified bioactive glass for functional coatings produced by thermal spray processing. Insights from this study support the optimization of APS-deposited radiopaque bioactive coatings for load-bearing implant applications and their future use in bone tissue engineering devices.

## 2 Materials and methods

### 2.1 Bi-BG feedstock powder fabrication

The feedstock used for APS deposition consisted of a melt-derived bismuth-containing bioactive glass (Bi-BG) synthesized following the procedure described in our previous work, with oxide contents (in wt. %) of 42.2 SiO_2_, 22.1 Na_2_O, 22.1 CaO, 5.6 P_2_O_5_, and 6.3 of Bi_2_O_3_. Mixed oxide precursors were melted in a PtRh10 furnace at 1450 °C for 3 h under ambient conditions. The molten glass was then quenched onto a cooled stainless-steel plate to prevent crystallization. The resulting glass was crushed, milled, and sieved to obtain a powder with a particle size ≤40 μm, suitable for plasma spraying.

### 2.2 Bi-BG coating fabrication by APS

**Fig. 1:**
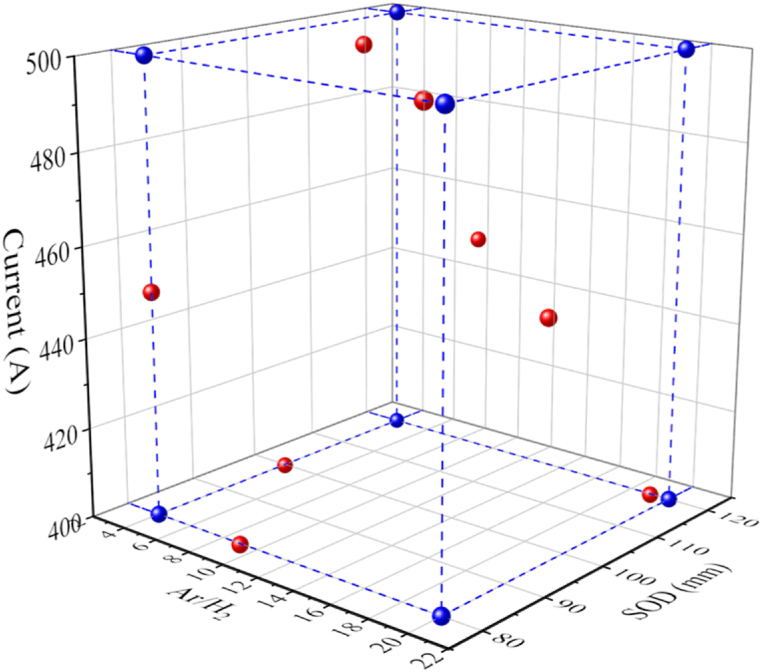
APS thermal spray parameter matrix. Blue dots represent the full experimental domain; red dots represent the selected combinations.

Coatings were deposited by APS thermal spray onto Ti grade 5 alloy substrates (2 × 2 cm, 0.3 mm thick). The substrates were first grit-blasted following the NBN EN 13507:2001 standard, using F36 Al_2_O_3_ particles (∼500 μm) at 6 bar, 100 mm distance, and a velocity of 75 mm/min to enhance surface roughness and adhesion. Subsequently, the substrates were ultrasonically cleaned in acetone for 10 min to remove residual Al_2_O_3_ and other contaminants. Bi-BG powder was radially fed into the F4 plasma gun operated with a fixed total gas flow of 8.6 standard liters per minute (SLPM) at 3 bars, a powder feed rate of 10 g/min, and a traverse speed of 1 m/s with a 4 mm step. Substrates were preheated to 300-350 °C, while cooling was provided by two compressed-air jets at 0.2 MPa during spraying. A Latin square experimental design was employed to study the influence of APS parameters. Three factors varied at three levels: plasma intensity (400, 450, and 500 A), SOD (80, 100, and 120 mm), and Ar/H_2_ plasma flow ratios (57/3, 54/6, 48/12). These parameters determined plasma net power and particle heating conditions, enabling systematic evaluation of their effects on Bi-BG coating morphology and structure. Fig. 1 illustrates the parameter matrix, where blue markers represent the experimental domain and red markers indicate the selected combinations. The resulting coatings labelled C1 - C9 with their corresponding deposition parameters are listed in Table 1.

**Table 1:** Atmospheric plasma spray parameters used for coatings C1 - C9.

	Ar (SLPM)	H_2_ (SLPM)	Stand off distance (mm)	Intensity (A)	Net power (kW)
**C1**	54	6	80	400	26.7
**C2**	57	3	80	500	30
**C3**	48	12	80	450	34.2
**C4**	57	3	100	450	26.8
**C5**	48	12	100	400	30.8
**C6**	54	6	100	500	32.8
**C7**	57	3	120	400	24.7
**C8**	54	6	120	450	29.9
**C9**	48	12	120	500	37.5

### 2.3 Characterization

#### 2.3.1 Bi-BG feedstock powder characterization

The Bi-BG feedstock powder was characterized for its particle size distribution (PSD), morphology, phase composition, and thermal properties. Particle size was measured by laser diffraction using the Fraunhofer model (Granulo LA-950, Horiba Scientific), under moderate stirring and sonication to prevent agglomeration. Morphology was analyzed by scanning electron microscopy (SEM, HITACHI TM3000) in backscattered mode at 15 kV. X-ray diffraction (XRD; Bruker D8 ADVANCE, USA), with monochromatic Cu Kα radiation (λ = 1.5406 Å), operated at 40 kV and 40 mA, scanned from 20° to 70° 2θ with a 0.02° step and 0.5 s counting time, was used to analyze the phase composition. Thermal behavior was evaluated by differential scanning calorimetry (TG–DSC STA 443F3, Netzsch) from 20 °C to 1300 °C in air at 5 °C/min. Characteristic glass transition (T_g_), crystallization (T_c_), and melting (T_m_) temperatures were identified from the corresponding endothermic and exothermic peaks.

#### 2.3.2 Bi-BG coatings characterization

##### 2.3.2.1 Surface structure and morphology

The coatings phase composition was examined by XRD under the conditions described in section 2.3.1. Surface and cross-sectional morphologies were examined by SEM. Energy-dispersive X-ray spectroscopy (EDS) was used to follow the bismuth distribution on the coating's surface using a Xmax^80^ EDS (Oxford) at 6 mm and 20 kV. Raman spectroscopy (RENISHAW inVia Reflex) was employed to assess the short-range structural order using a 532 nm excitation laser (28.5 mW) over the wavenumber range 500–1200 cm^−1^. Spectra were analyzed using a Voigt multi-peak deconvolution fitting procedure in AAnalyzer [28]. Peak fitting was performed by nonlinear least-squares minimization using a Levenberg–Marquardt optimization routine. For each spectrum, the peak position, intensity, and full width at half maximum were refined simultaneously, while the same initial band assignment and fitting strategy were applied to all coatings to ensure comparability [29]. The standard uncertainty associated with each fitted parameter was estimated from the covariance matrix of the fit [30]. Each spectrum was subjected to repeated independent peak-fitting analyses to evaluate the repeatability of the Raman peak-fitting procedure. Raman band positions were reported as average fitted positions across all coatings, with expanded uncertainties, U = 2u, corresponding approximately to a 95 % confidence level and including both fitting variability and sample-to-sample variation. In contrast, the uncertainties of the relative integrated intensities were calculated from the dispersion of the integrated peak areas obtained from the repeated independent fits and are also reported as expanded uncertainties, U = 2u, corresponding approximately to a 95 % confidence level; therefore, they represent fitting-related variability. Given the complex band overlap characteristic of multicomponent bioactive glasses, the fitted peak intensities are used as comparative indicators of structural changes across the coatings rather than absolute quantitative fractions of individual glass network units.

##### 2.3.2.2 Cross-section hardness, thickness, and porosity

Vickers microhardness was measured at the coating-substrate interface using a diamond pyramidal indenter (Leica VMHT10A) under a 50 gf load (15 s dwell), following ASTM C1327 to follow the coating-substrate cohesion [31,32]. Five measurements per sample were averaged. A 50x optical system attached to the micro-indenter improved measurement precision. Cross-sectional SEM micrographs were analyzed using ImageJ software (NIH) to determine coating thickness and porosity [14]. Porosity was quantified from 3 randomly selected cross-sectional images per sample. Image contrast and thresholding were adjusted to exclude polishing-induced cracks. The final porosity value for each coating corresponded to the mean of the 3 measurements.

### 2.4 Statistical analysis

A Latin Square experimental design was employed to statistically evaluate the influence of atmospheric plasma spray parameters on coating properties [33,34]. This design allows for determining the influence degree of Ar/H_2_, SOD, and arc current intensity at three levels following the matrix shown in Table 2. Analysis of Variance (ANOVA) was performed to determine the statistical significance of each factor on the measured Vickers microhardness, porosity, and coating thickness. The Latin Square design might be expressed as in Equation (1).

(1) $$Y_{ijk}=\mu +\alpha_{i}+\beta_{j}+\gamma_{k}+\varepsilon_{ijk}$$

Where:

**Table 2:** Latin Square matrix showing (a) the symbolic factors positions and (b) the selected APS processing parameter for coatings (C1–C9).

a)	Column Factor Intensity (β_j_)		
Row Factor SOD (α_i_)	β_1_	β_2_	β_3_
α_1_	γ_2_	γ_3_	γ_1_
α_2_	γ_3_	γ_1_	γ_2_
α_3_	γ_1_	γ_2_	γ_3_

b)	400 A (j = 1)	450 A (j = 2)	500 A (j = 3)
**80 mm (i=1)**	C1: 54/6 (k = 2)	C3: 48/12 (k = 1)	C2: 57/3 (k = 3)
**100 mm (i=2)**	C5: 48/12 (k = 1)	C4: 57/3 (k = 3)	C6: 54/6 (k = 2)
**120 mm (i=3)**	C7: 57/3 (k = 3)	C8: 54/6 (k = 2)	C9: 48/12 (k = 1)

$Y_{ijk}$ = Response measurement

μ = Response mean

$\alpha_{i}$ = SOD effect

$\beta_{j}$ = Intensity effect

$\gamma_{k}$ = Ar/H_2_ ratio effect

$\varepsilon_{ijk}$ = Residual error

The percentage contribution of each factor was determined using Equation (2).

(2) $$Factor\;contribution\;\left(\%\right)=\frac{Sum\;of\;squares\;for\;each\;factor\;\left(SSfactor\right)}{Total\;sum\;of\;squares\;\left(SStotal\right)}\times 100$$

## 3 Results and discussion

### 3.1 Bi-BG feedstock powder characterization

The Bi-BG feedstock powder used for plasma spray deposition corresponded to a bismuth-containing derivative of 45S5 BG, modified with 6.3 wt % Bi_2_O_3_. This composition was selected for its previously reported combination of amorphous character, biocompatibility, bioactivity, angiogenicity, and antibacterial performance, as well as the enhanced radiographic contrast imparted by Bi_2_O_3_ [26,27].

**Fig. 2:**
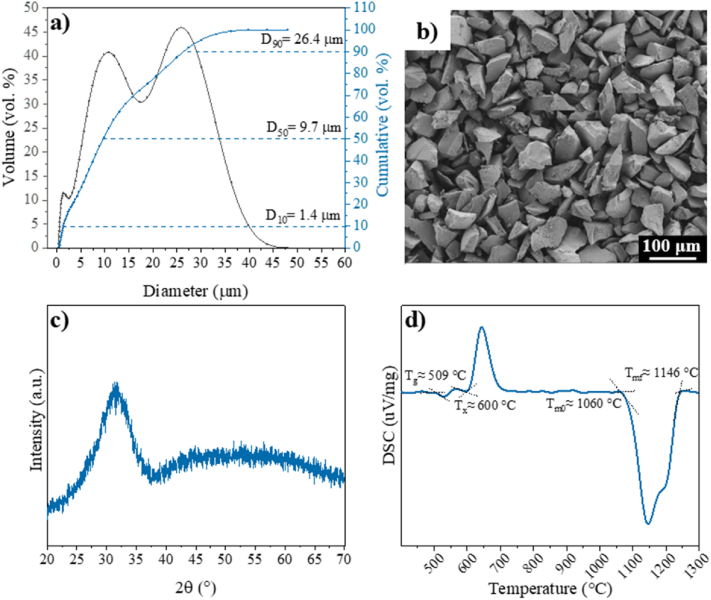
Characterization of Bi-BG feedstock powder to be used for APS coatings deposition. a) PSD, b) SEM micrograph, c) XRD diffractogram, and d) DSC curve.

The particle size of the Bi-BG (Fig. 2a) exhibited characteristic diameters of D_10_ ≈ 1.4 μm, D_50_ ≈ 9.7 μm, and D_90_ ≈ 26.4 μm. The coexistence of fine and coarse fractions suggests that particles may experience slightly different thermal histories when exposed to the high-enthalpy plasma jet. However, the relatively narrow distribution should ensure that most particles experience similar heating and melting conditions, favoring compositional and structural uniformity in the resulting coatings. SEM micrographs in Fig. 2b revealed angular and irregular particle morphologies with sharp edges and fractured surfaces, typical of brittle fracture in quenched glasses.

The XRD pattern of the Bi-BG powder (Fig. 2c) shows a broad diffuse shoulder centered around 25–35° 2θ and an absence of sharp diffraction maxima in the 20–70° 2θ range, confirming the amorphous nature of the glass. This is consistent with silicate-based bioactive glasses, in which structural ordering is limited to short-range Si-O-Si and P-O linkages, while long-range periodicity is absent [35,36]. The amorphous nature of the Bi-BG is advantageous because it avoids the need to fully melt and rapidly quench the material during plasma spraying to obtain an amorphous coating, a key structural requirement for a bioactive glass coating.

DSC analysis of the Bi-BG feedstock powder shown in Fig. 2d reveals a glass transition temperature T_g_ of approximately 509 °C, followed by the onset of crystallization (T_x_) at around 600 °C and a crystallization peak (T_c_) near 643 °C. The melting process began at approximately 1060 °C and was completed at 1146 °C, corresponding to the liquidus temperature of the glass. These thermal events are characteristic of silicate-based bioactive glasses but occur at slightly lower temperatures than those reported for 45S5 compositions [2], where T_g_ and T_c_ are typically higher. This downward shift is attributed to the presence of Bi_2_O_3_, which modifies the glass network through the formation of [BiO_3_] and [BiO_6_] units with a dual former-modifier role, effectively reducing network connectivity and, consequently, the rigidity and thermal resistance of the glass matrix [26]. The thermal stability window between T_g_ and T_x_ is approximately 91 °C, indicating a moderate tendency toward crystallization during heating. The presence of Bi-containing units may introduce localized compositional or structural heterogeneities that facilitate nucleation and promote devitrification, a factor that must be carefully considered when selecting APS parameters for Bi-BG coatings on Ti substrates [2]. Despite this, the Bi-BG thermal profile remains compatible with plasma spraying. The melting state of in-flight particles allows sufficient softening without requiring complete melting, which favors the retention of an amorphous structure in the deposited coatings. Preserving the amorphous nature of glass is essential for maintaining its functional performance, particularly its bioactivity and dissolution-driven surface reactivity. Overall, the Bi-BG feedstock powder combines a suitable particle-size distribution with a fully amorphous structure and thermal properties suitable for APS processing.

### 3.2 Bi-BG coatings characterization

#### 3.2.1 Coatings structural analyses

##### 3.2.1.1 Surface phase analysis of Bi-BG coatings

**Fig. 3:**
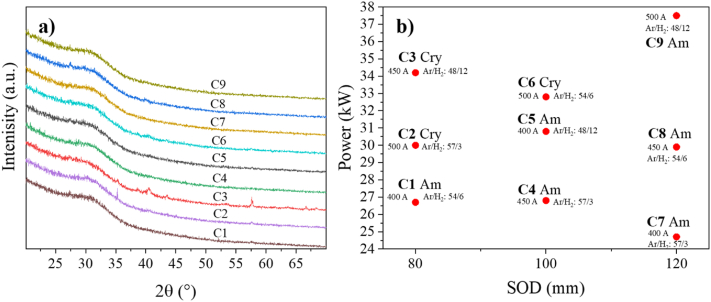
(a) X-ray diffraction patterns of coatings C1 to C9 deposited under varying APS parameters and (b) process parameter map showing the relationship between SOD, plasma net power, and the appearance of substrate-related crystalline reflection.

The structural response of Bi-BG under APS conditions was evaluated by XRD to assess the phase evolution of coatings C1-C9 as a function of stand-off distance and plasma net power. Fig. 3a shows the diffractogram of the coatings, while Fig. 3b maps the process parameters in terms of SOD and plasma net power, correlating them with the crystallization peaks from the substrate.

Most of the coatings exhibit a broad diffraction halo in the 25°–35° 2θ range, indicative of an amorphous (Am) silicate network. This feature reflects the absence of a long-range atomic order and is typical of bioactive glasses, whose disordered structure supports ion release and bioactivity.

Coatings C1, C2, and C3 were sprayed at a short SOD of 80 mm using net power inputs of 26.7, 30.0, and 34.2 kW, respectively. Under these conditions, C2 and C3 showed substrate-related crystalline reflections at 35.2, 38.6, 40.6, 43.5, and 57.6° 2θ. These reflections suggest that the coating layer was locally thinner, less continuous, or less effective in attenuating the Ti substrate signal under these spraying conditions. When SOD increased to 100 mm, C4 and C5 retained the amorphous response of the Bi-BG feedstock. However, C6, deposited at the highest plasma net power within the 100 mm SOD series, showed weak reflections at the same 2θ positions, suggesting a local reduction in coating continuity or thickness. At 120 mm SOD, C7, C8, and C9 exhibited predominantly amorphous halos across net power values from 24.7 to 37.5 kW. Even for C9, deposited at the highest net power, the longer SOD preserved the amorphous XRD response and suppressed clear substrate contributions. These results indicate that SOD plays a dominant role in preserving the amorphous coating response and maintaining sufficient surface coverage during APS processing.

The process map in Fig. 3b further illustrates the relationship between the APS and the appearance of substrate-related reflections. These reflections appear mainly at short SOD combined with higher plasma net power, as observed for C2 and C3, and reappear weakly in C6 at intermediate SOD and high net power. This behavior can be rationalized by considering the thermal and kinetic history of the in-flight particles. At short SOD, particles have limited residence time in the plasma jet, which can restrict homogeneous melting and stable splat consolidation. When this condition is combined with high plasma net power, localized overheating, volatilization, or inefficient deposition may reduce coating continuity and allow the substrate signal to become detectable. Such volatilization might also be related to the detected globular porosity observed in the coatings' cross-section. At the same short SOD but lower net power, as in C1, substrate reflections are not evident, suggesting better surface coverage within the XRD probing depth. Overall, the XRD results indicate that longer SOD favors a more continuous amorphous coating response, while excessive thermal input can locally compromise coating uniformity. The presence of Ti substrate peaks reflects increased X-ray penetration through thinner coatings, which, in this study, is a direct consequence of reduced deposition efficiency under the corresponding APS processing conditions. Since all other variables, such as powder feed rate, substrate roughness, and number of spray passes, were held constant, coating thickness and deposition efficiency are strongly coupled, making substrate peak intensity a valid indirect indicator of coating completeness.

##### 3.2.1.2 Surface structural and Bi distribution analyses of coatings

**Fig. 4:**
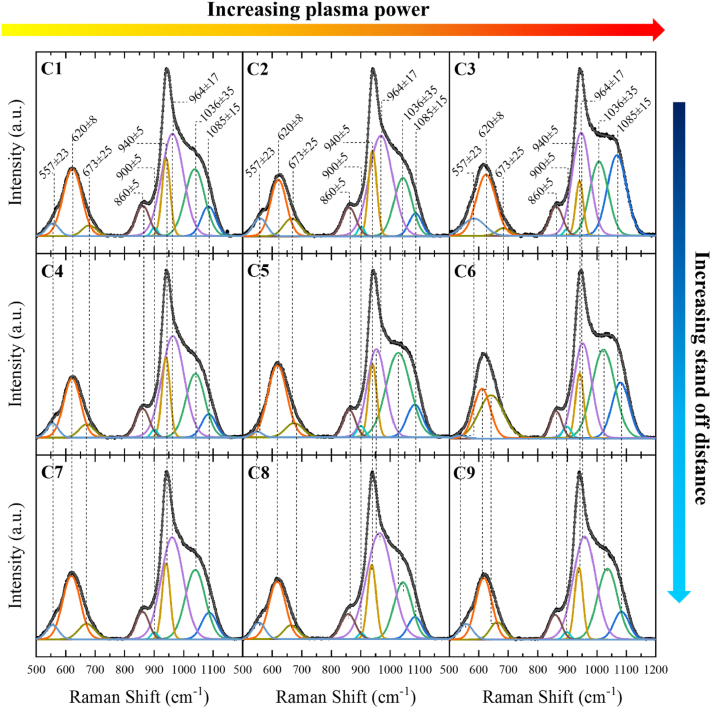
Raman peak-fitting analysis of Bi-BG coatings C1–C9 deposited under different APS plasma power and stand-off distance conditions. The fitted bands represent vibrational contributions used to compare short-range structural changes among coatings.

While XRD establishes the predominantly amorphous character of the coatings and reveals differences in substrate signal attenuation, Raman spectroscopy provides complementary information on short-range structural rearrangements within the amorphous glass network. The Raman spectra of the Bi-BG coatings (Fig. 4) show that the glass network structure is largely preserved after APS processing, although the relative contributions of the main vibrational bands vary with the deposition condition. The spectra are consistent with a multicomponent silicate glass network in which Bi, Si, and P participate in the short-range structure. A prominent band at approximately 557 ± 23 cm^−1^ is assigned to Bi–O stretching vibration in [BiO_6_] octahedral units, which is a characteristic of bismuth-containing vitreous networks [37,38]. The band at 620 ± 8 cm^−1^ is associated with the rocking motion of non-bridging oxygen atoms (NBOs) in the silicate network [39,40], whereas the contribution at 673 ± 25 cm^−1^ corresponds to bending vibrations of bridging oxygens (BOs) in Si-O-Si linkages [41,42]. These bands agree with the vibrational features expected for modified 45S5-type bioactive glasses [26].

In the mid-wavenumber region between 850 and 950 cm^−1^, several overlapping bands reflect contributions from different silicate and bismuth-containing environments. The band near 860 ± 5 cm^−1^ is associated with the symmetric stretching of isolated orthosilicate [SiO_4_]^4-^ units [41,43], suggesting a certain degree of silicate network depolymerization. A shoulder around 900 ± 5 cm^−1^ is attributed to Bi–O vibrations in pyramidal [BiO_3_] units, indicating the coexistence of multiple Bi coordination states within glass [44,45].

The band at 940 ± 5 cm^−1^ is assigned to symmetric stretching of metasilicate [SiO_3_]^2-^ units in chains or rings, consistent with an intermediate degree of silicate polymerization. The band near 964 ± 17 cm^−1^ corresponds to the symmetric stretching of P–O–P linkages in phosphate groups embedded in the silicate network [42]. The high-intensity bands at 1036 ± 35 and 1085 ± 15 cm^−1^ are assigned to asymmetric Si–O–Si stretching vibrations. The lower wavenumber 1036 ± 35 cm^−1^ band is associated with Bi-modified silicate environments, where modifier-related perturbation of the silicate network favors more depolymerized or Bi-enriched local structures. In contrast, 1085 ± 15 cm^−1^ is associated with less modified silicate domains that retain a higher degree of network polymerization. Table 3 summarizes the complete band assignment and the corresponding relative integrated intensities.

**Table 3:** Raman band assignments and relative intensities for coatings C1–C9. Band positions are reported in cm^−1^ as average fitted positions ± expanded uncertainty. Values inside the table correspond to relative integrated intensity (%) ± expanded uncertainty, calculated from independent peak-fitting results and corresponding approximately to a 95 % confidence level.

Coating	557 ± 23 cm^−1^ Bi–O in [BiO_6_] units	620 ± 8 cm^−1^ NBOs	673 ± 25 cm^−1^ BOs	860 ± 5 cm^−1^ [SiO_4_]^4-^ units	900 ± 5 cm^−1^ Bi–O in [BiO_3_] units	940 ± 5 cm^−1^ [SiO_3_]^2-^ units	964 ± 17 cm^−1^ P–O–P	1036 ± 35 cm^−1^ Bi-modified Si–O–Si units	1085 ± 15 cm^−1^ Si–O–Si units
**C1**	2.3 ± 0.3	19.3 ± 1.4	1.9 ± 1.1	6.2 ± 0.3	1.1 ± 0.3	11 ± 2	34 ± 5	18 ± 5	6 ± 2
**C2**	4.1 ± 0.4	14 ± 3	4 ± 3	6.4 ± 0.3	1.2 ± 0.3	13 ± 3	37 ± 1	16 ± 3	4 ± 2
**C3**	5.4 ± 0.7	16.3 ± 0.4	1.3 ± 0.3	5.1 ± 0.6	0.5 ± 0.1	6 ± 2	28 ± 7	15 ± 5	22 ± 2
**C4**	2.6 ± 0.1	16 ± 2	3 ± 2	6.3 ± 0.2	1.2 ± 0.5	12 ± 3	36 ± 2	18 ± 4	5 ± 2
**C5**	0.8 ± 0.3	20 ± 2	3 ± 2	4 ± 2	2.4 ± 1.5	10 ± 2	25 ± 5	25 ± 7	8 ± 4
**C6**	0.4 ± 0.1	14 ± 6	11 ± 6	4 ± 2	2.4 ± 1.2	8 ± 3	23 ± 5	25 ± 4	12 ± 5
**C7**	2.6 ± 0.2	17 ± 3	4 ± 3	5.3 ± 0.5	1.7 ± 1.5	10 ± 2	33 ± 3	21 ± 1	6 ± 1
**C8**	3.3 ± 0.1	14 ± 1	4 ± 1	5.4 ± 0.8	0.5 ± 0.3	9.6 ± 0.9	43 ± 2	16 ± 2	4 ± 2
**C9**	3.1 ± 0.5	16 ± 4	3 ± 3	4 ± 2	3 ± 2	10 ± 2	35 ± 2	20 ± 4	6 ± 2

**Fig. 5:**
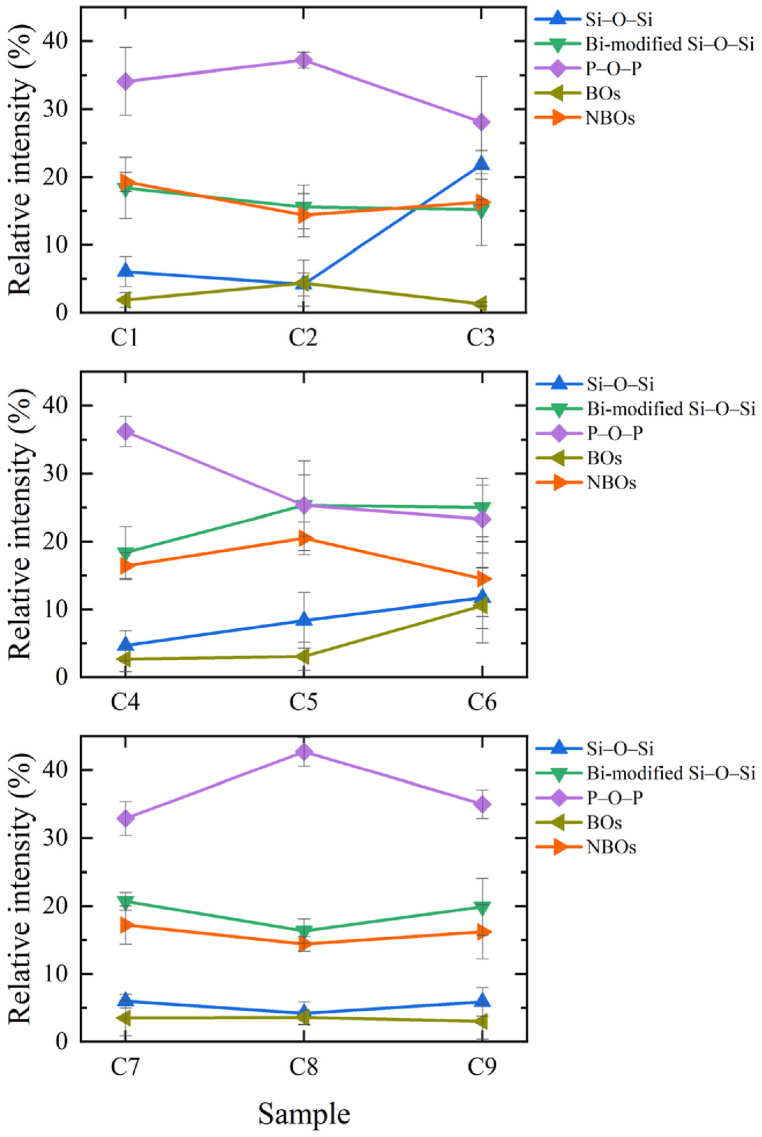
Relative intensity profiles of selected Raman bands across coatings C1 to C9 deposited under varying APS parameters. These bands were selected based on their dynamic variation across samples and their significance for tracking changes in network polymerization and modifier incorporation.

Fig. 5 presents the relative integrated intensities of five selected Raman bands across coatings C1 to C9, with the corresponding expanded uncertainties to identify the Raman features most responsive to the APS process parameters. These bands were selected because they show relevant variation among the coatings and because they are structurally meaningful for tracking silicate network polymerization, phosphate-related units, and Bi-induced modification. The selected contributions include a band near 964 cm^−1^, primarily associated with P–O–P stretching in phosphate-rich environments, a band at 1036 cm^−1^ assigned to asymmetric Si–O–Si stretching in Bi-modified regions, a band at 1085 cm^−1^ attributed to asymmetric Si–O–Si stretching in less modified regions, a band at 673 cm^−1^ associated mainly with BO bending, and a band at 620 cm^−1^ related predominantly to NBO rocking modes.

The P–O–P related contribution near 964 cm^−1^ shows the clearest variation among the selected bands. Coating C8 exhibits the highest relative integrated intensity, 43 ± 2 %, indicating the strongest phosphate-related Raman contribution under the corresponding APS condition. C2, C4, C7, and C9 also retain comparatively high P–O–P contributions, although some of these values overlap within their uncertainty ranges. This behavior is consistent with greater retention or stabilization of phosphate-related environments when the APS conditions provide sufficient particle heating while limiting excessive thermal restructuring. Under these conditions, the glass network may better preserve P–O–P linkages and reduce the redistribution or partial volatilization of thermally labile P_2_O_5_. In contrast, C5 and C6 show lower P–O–P contributions, 25 ± 5 % and 23 ± 5 %, respectively, suggesting structural rearrangements and/or partial loss of phosphate-related species during APS processing.

The Bi-modified Si–O–Si contribution near 1036 cm^−1^ remains prominent in all coatings, indicating that Bi-modified silicate environments persist after plasma spraying. This contribution is associated with silicate regions affected by Bi_2_O_3_ incorporation, where Bi-related modifier behavior can weaken Si–O connectivity and perturb silicate polymerization. The highest values occur in C5 and C6, with 25 ± 7 % and 25 ± 4 %, respectively. Although some uncertainty ranges overlap, the increase at intermediate SOD suggests that these conditions favor local rearrangement of Bi-containing silicate environments, likely through enhanced cation mobility during particle heating. On the contrary, the contribution near 1085 cm^−1^ increases markedly in C3, reaching 22 ± 2 %, which is clearly higher than the values observed for most other coatings. C6 also shows an elevated central value of 12 ± 5 %, although its broader uncertainty warrants a more cautious interpretation. This trend suggests that high plasma power can promote structural rearrangement toward more polymerized Si–O–Si environments, but the effect depends on the SOD. In C3, the combination of high plasma power and short SOD likely increases particle temperature while limiting in-flight cooling before impact, favoring the retention of more connected Si–O–Si environments. In C6, the high plasma power at intermediate SOD may also promote cation mobility and local bond rearrangement, although the uncertainty prevents a strict quantitative separation from several other coatings. Therefore, the enhanced 1085 cm^−1^ contribution supports partial silicate repolymerization under high thermal input rather than a simple monotonic dependence on SOD or plasma power.

The BO bending contribution near 673 cm^−1^ provides additional support for partial silicate repolymerization, although its larger uncertainty requires cautious interpretation. C6 shows the highest central BO contribution, 11 ± 6 %, whereas most other coatings remain below approximately 4 %. This value should not be interpreted as a strict quantitative separation from all coatings, considering that the expanded uncertainty for C6 is relatively large. Nevertheless, the simultaneous increase in the BO contribution and the elevated 1085 cm^−1^ Si–O–Si contribution in C6 is consistent with the formation of more connected silicate environments under higher thermal input at intermediate SOD.

The NBO contribution near 620 cm^−1^ remains evident in all coatings, indicating that the coatings retain depolymerized silicate environments typical of modified bioactive glasses. The highest central values are observed for C1 and C5, with 19.3 ± 1.4 % and 20 ± 2 %, respectively, whereas C2, C6, and C8 show lower central values. A higher NBO contribution is consistent with lower silicate network connectivity, typically promoted by modifier oxides. Conversely, stronger thermal exposure can favor local bond reorganization and partial Si–O–Si re-formation, reducing the NBO-related Raman contribution, as suggested by the lower central value in C6. However, the uncertainty ranges, particularly for C6 and C9, indicate that the NBO contribution does not follow a simple monotonic trend with plasma power or SOD. Instead, the NBO evolution reflects a balance between modifier-induced depolymerization, local cation redistribution, and thermally driven silicate reorganization during APS deposition.

The Raman intensity trends indicate that APS parameters modify the short-range structure of Bi-BG coatings through a balance among phosphate retention or redistribution, Bi-modified silicate environments, and partial silicate repolymerization under higher thermal input. Short SOD combined with high plasma power, as in C3, favors a stronger contribution from more polymerized Si–O–Si environments. This structural shift may reduce dissolution-driven reactivity by decreasing the fraction of readily exchangeable depolymerized silicate units. Intermediate SOD combined with higher thermal input, as in C5 and C6, enhances the contribution from Bi-modified silicate environments but reduces the phosphate-related P–O–P contribution, suggesting stronger phosphate redistribution and/or partial loss of phosphate-related species during APS processing. In contrast, the 120 mm SOD condition, especially C8, preserves a strong P–O–P contribution while maintaining Bi-modified and depolymerized silicate features.

**Fig. 6:**
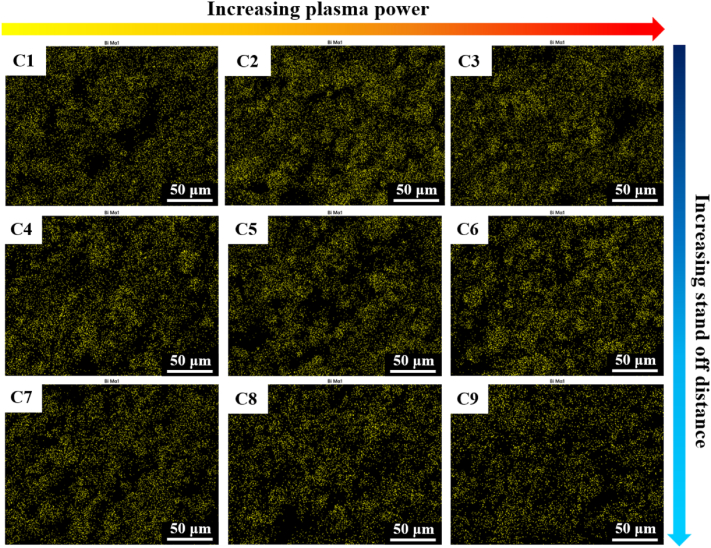
EDS Bi Mα1 elemental maps of coatings C1–C9, confirming homogeneous bismuth distribution across the coating surface as a function of increasing plasma net power and SOD.

C7 and C8 provide the most favorable Raman response among the studied conditions. C8 shows the clearest retention of the phosphate-related contribution, whereas C7 maintains a balanced contribution from phosphate, Bi-modified silicate, and NBO-related environments. Together with the amorphous character observed by XRD, these Raman results support arc currents around 400 – 450 A and a SOD of 120 mm as suitable conditions for preserving the glassy short-range structure of Bi-BG coatings while limiting excessive thermal restructuring. These conditions likely support a semi-molten state of in-flight particles followed by rapid solidification upon impact, resulting in predominantly glassy coatings with retained depolymerized features and minimized crystallization. EDS elemental mapping of Bi Mα1 emission was performed across all nine coatings to confirm bismuth retention and spatial distribution after APS processing (see Fig. 6). The elemental characterization reveals a homogeneous bismuth distribution throughout the coating surface in all conditions, with no evidence of preferential segregation or depletion zones. The uniform signal intensity observed across the processing window, regardless of plasma net power or SOD, confirms that Bi is retained within the glass network after thermal spraying and is not selectively volatilized under the tested APS conditions.

##### 3.2.1.3 Surface and cross-section morphology of coatings

**Fig. 7:**
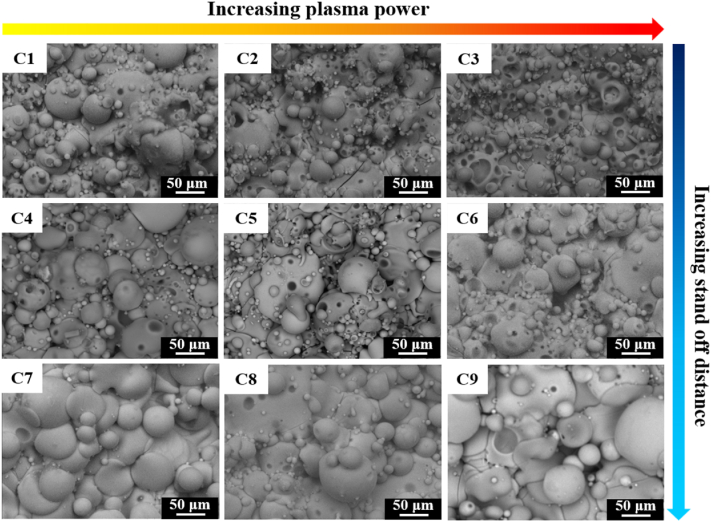
Top-view SEM micrographs of coatings as a function of the APS net power and SOD.

**Fig. 8:**
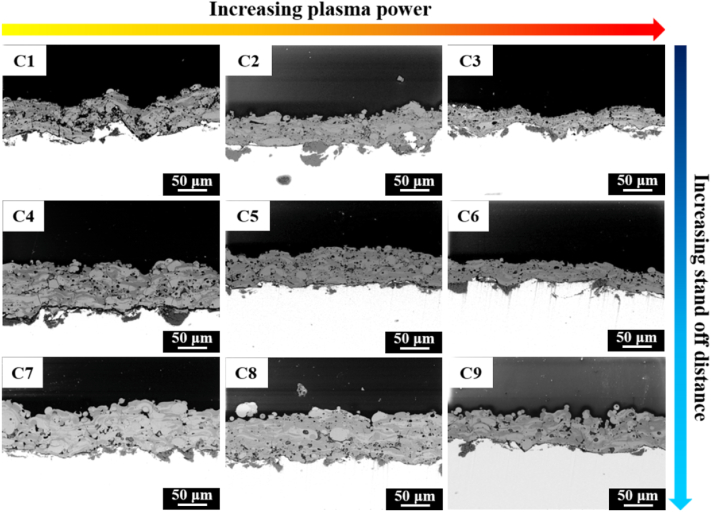
Cross-section SEM micrographs of coatings as a function of the APS net power and SOD.

Surface and cross-section morphology of coatings were examined to correlate APS parameters with particle melting, splat formation, porosity, and coating cohesion. Fig. 7, Fig. 8 show representative surface and cross-sectional SEM micrographs, respectively, for coatings C1 – C9.

The top-view images in Fig. 7 reveal distinct differences in surface morphology across the processing window. Coatings C1 to C2, deposited at the shortest SOD of 80 mm, exhibit irregular surfaces containing partially melted particles, fragmented debris, and numerous spherical features, indicating insufficient particle melting due to limited in-flight residence time and thermal exposure at the applied plasma net powers. As a result, splat spreading is incomplete, leading to a rough and porous surface. In contrast, coating C3, sprayed at a higher plasma net power but the same short SOD, shows evidence of a higher in-flight melting degree in the particle surface, manifested by bubble formation and spherical surface cavities that are consistent with volatilization of glass constituents and subsequent gas release during solidification, as previously reported for APS bioactive glasses [14,15]. The surface morphology at intermediate SOD in coatings C4 to C6 becomes more refined. Coatings C4 and C5 exhibit larger, well-flattened splats and smoother coverage, indicating improved particle melting and spreading behavior. However, coating C6 still presents irregular features and some unmelted fragments, suggesting that the combination of higher net power and intermediate SOD may induce unstable particle heating or localized overheating, which can compromise melting uniformity and splat formation. The coatings sprayed at the longest SOD of 120 mm C7 to C9 exhibit the smoothest and most homogenous surfaces. C8 shows extensive flattened splats with minimal unmelted particles or fine debris, reflecting an effective balance between sufficient semi-molten particle state and extended flight time, allowing the Bi-BG to reach an optimal viscosity for deformation and bonding on impact. Coating C9 also appears relatively smooth, but the presence of larger isolated particles and local textural inhomogeneities suggests possible overspreading or gas entrapment associated with the combination of high plasma net power and long in-flight path.

The cross-sectional SEM micrographs in Fig. 8 corroborate and extend the surface observations. Coatings C1 to C3 appear relatively porous and non-uniform, with visible interlamellar voids and intersplat cohesion, particularly in C1 and C2, where the weak bonding indicates that many particles were insufficiently softened at impact. Although C3 benefits from higher net power, the short SOD still constrains cooling and solidification dynamics, resulting in only moderate improvement in structural integrity. In the intermediate SOD group, coatings C4 and C5 show more continuous lamellae, reduced porosity, and better-defined interfaces, consistent with more efficient melting and spreading during deposition. Coating C6, however, reveals internal defects and vertically oriented pores that may arise from vapor entrapment or localized volatilization of thermally sensitive species such as P_2_O_5_ during spraying, phenomena that are known to occur under high net power plasma conditions [14,15].

C7 and C8 coatings, deposited at a SOD of 120 mm, exhibit a relatively thick and compact microstructure, with well-formed intersplat lamellae and minimal defects, reducing the coating porosity.

Coating C9, also sprayed at 120 mm SOD, shows slightly lower thickness and a more heterogeneous internal morphology compared to C7 and C8. Occasional voids and indications of reduced deposition efficiency are visible, which can be associated with the combination of maximum net power and extended flight time, conditions that may cause excessive particle velocity, localized overheating, partial volatilization, or oxidation, thus lowering deposition yield.

Overall, the surface and cross-sectional observations support the fact that, as expected, the interaction between SOD and plasma net power has a critical impact on coating quality. Processing conditions that ensure adequate particle surface melting followed by longer particle in-flight time, such as those used for C7 and C8, favor the development of a well-overlapped splats structure with reduced porosity. In contrast, excessive thermal input, even at long SOD, as in C9, can compromise coating thickness and introduce structural heterogeneities. These results underscore the importance of carefully tuning plasma net power and SOD. Higher SOD combined with moderate-to-high net power promotes improved particle melting, more uniform splat morphology, and lower porosity, which are key factors for achieving coatings with robust mechanical performance and coating structural integrity.

##### 3.2.1.4 Cross-section hardness, porosity, and thickness of coatings

**Fig. 9:**
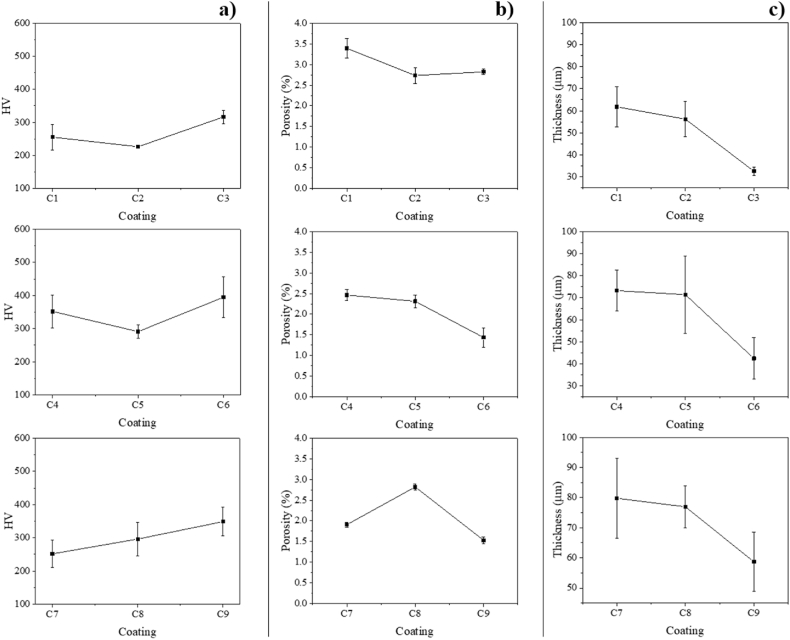
Cross-section a) hardness at the coating-substrate interface (HV_0.5_), b) porosity, and c) thickness.

Fig. 9 summarizes the hardness measured at the substrate-coating interface, porosity, and thickness of coatings C1–C9, providing insight into coating-substrate interfacial cohesion. The hardness values range from 251 ± 42 to 394 ± 61 HV_0.5_ and generally increase with plasma net power (see Fig. 9a). Coatings produced at 80 mm show lower hardness (255 - 316 HV_0.5_), indicating limited in-flight particle softening and less effective intersplat cohesion during build-up. In contrast, coatings deposited at 100 and 120 mm combined with higher plasma net power reach hardness values of ∼380 – 400 HV_0.5_, consistent with a more favorable semi-molten state, better splat deformation, and improved densification at the coating-substrate interface. The longer in-flight distance enhances the stability of particle heating and impact conditions, promoting improved stacking and interfacial compaction.

Porosity trends (Fig. 9b) are in agreement with these observations. Coatings sprayed at short SOD present the highest porosity, consistent with incomplete melting and intersplat gaps observed in cross-sectional SEM. Porosity decreases with increasing SOD and adequate plasma net power, reaching ∼2.3 – 2.5 % at 100 mm and dropping to ∼1.5 % under high-net power and long-distance conditions. This reduction documents more complete melting and splat spreading, which minimizes void formation and enhances microstructural homogeneity. The inverse relationship between hardness and porosity confirms the central role of densification in improving interfacial mechanical response. Coating thicknesses (Fig. 9c) are ∼55 – 65 μm at short SOD (C1, C2), whereas C3 shows a reduced thickness of ∼35 μm, likely due to reduced deposition efficiency under high net power and short flight distance. At 100 mm SOD, the thickness stabilizes around 70–75 μm, indicating more efficient buildup. The maximum thickness is obtained for C7, reflecting an optimal balance between particle melting and impact conditions. At very high net power, thickness decreases again, suggesting overspreading, rebound of over-heated particles, and/or partial volatilization, which limit net mass deposition. Overall, the results demonstrate that increasing plasma net power, when combined with sufficient SOD, enhances intersplat formation, reduces porosity, and increases the interfacial hardness. However, excessive thermal input can detrimentally affect coating thickness and deposition efficiency, highlighting the need to optimize plasma net power rather than maximize it.

#### 3.2.2 Statistical analysis of the influence of APS parameters on the coating properties

Based on the summarized experimental results shown in Table 4, ANOVA was performed as described in Section 2.4 to quantify the individual contribution of each processing parameter influence in coating of selected features. Table 5 presents the contribution factor for all three response variables.

**Table 4:** Summarized results of Bi-BG coatings deposited under APS parameters selected in the Latin Square design**.**

Coating	Ar/H_2_ Ratio (SLPM)	SOD (mm)	Intensity (A)	Hardness (HV_0.5_)	Porosity (%)	Thickness (μm)
**C1**	54/6	80	400	255.45 ± 38.67	3.39 ± 0.23	61.80 ± 9.09
**C2**	57/3	80	500	226.55 ± 2.60	2.74 ± 0.19	56.20 ± 7.98
**C3**	48/12	80	450	316.54 ± 20.20	2.82 ± 0.06	32.60 ± 1.95
**C4**	57/3	100	450	352.13 ± 49.29	2.46 ± 0.13	73.20 ± 9.31
**C5**	48/12	100	400	291.18 ± 19.50	2.31 ± 0.15	71.40 ± 17.54
**C6**	54/6	100	500	394.87 ± 61.10	1.44 ± 0.23	42.40 ± 9.45
**C7**	57/3	120	400	251.59 ± 42.10	1.91 ± 0.06	79.80 ± 13.37
**C8**	54/6	120	450	296.16 ± 50.43	2.82 ± 0.07	77.00 ± 7.04
**C9**	48/12	120	500	348.64 ± 42.68	1.53 ± 0.08	58.67 ± 9.87
**Grand Mean**				**303.68**	**2.38**	**61.45**

**Table 5:** Factor contributions to coating properties from ANOVA (%).

	Hardness	Porosity	Thickness
**SOD**	44.61	52.73	31.50
**Intensity**	23.64	33.73	23.19
**Ar/H_2_**	17.58	8.83	15.99
**Error**	14.17	4.71	29.32
**R^2^**	**0.886**	**0.953**	**0.706**

**Fig. 10:**
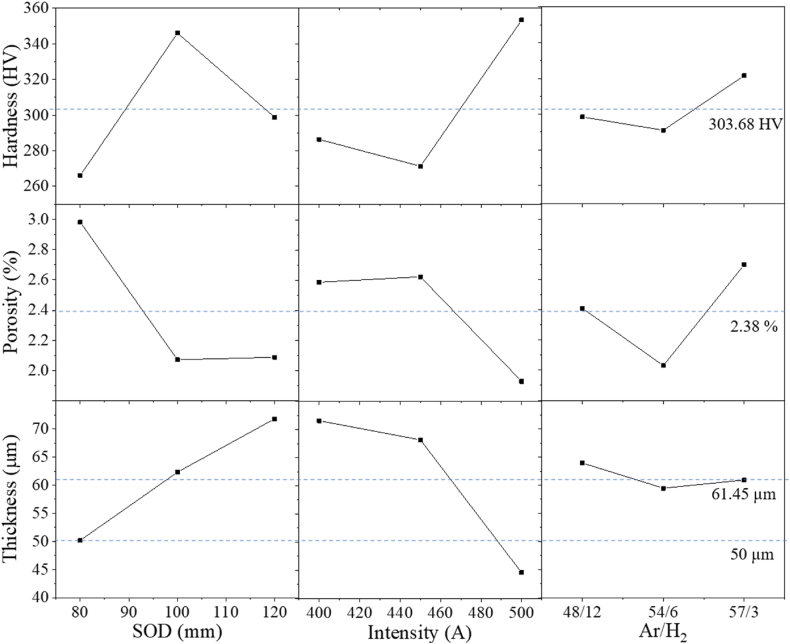
Main effects plots of means for hardness (HV_0.5_), porosity (%), and coating thickness (μm) means as a function of SOD, arc current intensity, and Ar/H_2_ gas flow rate ratio. Inserted lines indicate the grand mean for each response variable and the suggested ISO 13779-2:2018 minimum thickness threshold.

Table 5 presents the percentage contribution of each processing parameter to the total variance of hardness, porosity, and thickness derived from the Latin Square ANOVA, along with the residual error and the coefficient of determination R^2^. SOD showed the strongest influence across all responses, ranging from 31.50 to 52.73 %, affecting in-flight particle cooling time, melting state, velocity, and splat formation at impact. Arc current intensity was the second most influential factor, with 33.73 % influence, mainly on porosity, as expected, since intensity determines plasma enthalpy and, hence, the in-flight particle melting state. The Ar/H_2_ ratio showed lower contributions in coating/substrate interlayer hardness, porosity, and thickness, indicating a secondary effect within the tested range. The low residual error for porosity and hardness suggests that APS parameter interactions are negligible for these responses. The coating properties are predominantly governed by the independent effects of key process, particularly the molten-state particles and the impact velocity on the substrate, rather than by their combined interactions [46]. The thickness error of 29.32 % indicates that additional factors not included in this study, such as substrate roughness, in-flight particle velocity, and temperature, also significantly influence this response and might be considered in future work [47,48]. Fig. 10 presents the main effects plots for hardness, porosity, and thickness. SOD and plasma intensity exhibited clear and consistent effects across all three responses, whereas the Ar/H_2_ ratio showed comparatively negligible effects. SOD showed a non-monotonic effect for hardness, with a desired response at 100 mm, while plasma intensity demonstrated a progressive increase toward higher current levels, producing the highest thickness across all factors and levels at 500 A, suggesting the strong influence of the plasma enthalpy on the splat formation and hardness changes upon in-flight particle impact. SOD has the strongest reducing effect on porosity, with the most significant change between 80 mm and 100 mm, indicating a steady porosity at higher spray distances. Plasma intensity supported this trend, and the porosity mean was the lowest at 500 A, while it remained comparable at 400 A and 450 A. A threshold effect at high current rather than a gradual linear response. Coating thickness increased monotonically with SOD, as greater spray distances allowed particles to progressively cool and reach an in-flight melting state before impact, thereby promoting splat formation and overlap. On the other hand, coating thickness decreased at high plasma intensity due to possible splashing effects from high molten-particle density, triggering splat splashing, satellite formation, and reduced deposition efficiency.

## 4 Conclusions

A systematic analysis of APS conditions enables the tailored fabrication of amorphous bismuth-containing 45S5 BG coatings. SOD was the most influential spray parameter in controlling particle thermal history and final coating structure. Statistical analysis confirmed that SOD influences 31 – 53 % of the total variance across hardness, porosity, and thickness, with arc current intensity as the second most influential factor. Long spray SOD enhances coating efficiency and improves splat formation and overlapping. Simultaneous mechanisms of particle in-flight melting state at high plasma enthalpy result in reduced coating thickness, highlighting the relevance of the plasma net power in the coating deposition efficiency. Raman spectroscopy revealed significant differences in the relative intensities of P–O–P and Si–O–Si vibrations, reflecting variations in the network polymerization. In particular, coatings deposited at SOD of 120 mm and plasma net powers around 24.7 – 29.9 kW provided the most favorable combination of structural integrity, homogeneous surfaces, compact cross-sections with low porosity, and Raman signatures indicative of depolymerized silicate and phosphate species consistent with structural preservation of the glass network after APS processing. The findings highlight that bismuth-modified 45S5 coatings respond to APS parameters differently than conventional bioactive glass systems, due to the composition-specific thermal behavior introduced by Bi_2_O_3_, and that balancing melting efficiency, quenching rate, and structural preservation is critical to obtain APS thermal spray radiopaque coatings to functionalize the surface of biomedical devices.

## CRediT authorship contribution statement

**G.A. Clavijo-Mejía:** Conceptualization, Data curation, Formal analysis, Funding acquisition, Investigation, Methodology, Project administration, Resources, Software, Supervision, Validation, Visualization, Writing – original draft, Writing – review & editing. **M. Michálek:** Formal analysis, Validation, Writing – review & editing. **F. Kurtuldu:** Investigation, Writing – review & editing. **L. Youssef:** Investigation, Methodology, Resources, Writing – review & editing. **E. Bussy:** Formal analysis, Investigation, Methodology. **A. Denoirjean:** Funding acquisition, Resources. **A. Magnaudeix:** Investigation, Validation. **D. Cabrera-German:** Formal analysis, Visualization, Writing – review & editing. **D. Galusek:** Resources, Writing – review & editing. **A.R. Boccaccini:** Investigation, Validation, Writing – review & editing.

## Declaration of competing interest

The authors declare the following financial interests/personal relationships which may be considered as potential competing interests:German A. Clavijo-Mejia reports financial support was provided by European Union. If there are other authors, they declare that they have no known competing financial interests or personal relationships that could have appeared to influence the work reported in this paper.

## Data Availability

Data will be made available on request.
